# Evidence for isolated evolution of deep-sea ciliate communities through geological separation and environmental selection

**DOI:** 10.1186/1471-2180-13-150

**Published:** 2013-07-08

**Authors:** Alexandra Stock, Virginia Edgcomb, William Orsi, Sabine Filker, Hans-Werner Breiner, Michail M Yakimov, Thorsten Stoeck

**Affiliations:** 1 University of Kaiserslautern, School of Biology, Erwin-Schroedinger-Str. 14, D-67663 Kaiserslautern, Germany; 2 Department of Geology and Geophysics, Woods Hole Oceanographic Institution, Woods Hole, MA 02543, USA; 3 Institute for Coastal Marine Environment, IAMC-CNR, Spianata S. Raineri, 86, 98122 Messina, Italy

**Keywords:** Ciliates, Hypersaline, Deep-sea anoxic basins, DHABs, Brine, Species sorting, Environmental filtering, Niche separation

## Abstract

**Background:**

Deep hypersaline anoxic basins (DHABs) are isolated habitats at the bottom of the eastern Mediterranean Sea, which originate from the ancient dissolution of Messinian evaporites. The different basins have recruited their original biota from the same source, but their geological evolution eventually constituted sharp environmental barriers, restricting genetic exchange between the individual basins. Therefore, DHABs are unique model systems to assess the effect of geological events and environmental conditions on the evolution and diversification of protistan plankton. Here, we examine evidence for isolated evolution of unicellular eukaryote protistan plankton communities driven by geological separation and environmental selection. We specifically focused on ciliated protists as a major component of protistan DHAB plankton by pyrosequencing the hypervariable V4 fragment of the small subunit ribosomal RNA. Geospatial distributions and responses of marine ciliates to differential hydrochemistries suggest strong physical and chemical barriers to dispersal that influence the evolution of this plankton group.

**Results:**

Ciliate communities in the brines of four investigated DHABs are distinctively different from ciliate communities in the interfaces (haloclines) immediately above the brines. While the interface ciliate communities from different sites are relatively similar to each other, the brine ciliate communities are significantly different between sites. We found no distance-decay relationship, and canonical correspondence analyses identified oxygen and sodium as most important hydrochemical parameters explaining the partitioning of diversity between interface and brine ciliate communities. However, none of the analyzed hydrochemical parameters explained the significant differences between brine ciliate communities in different basins.

**Conclusions:**

Our data indicate a frequent genetic exchange in the deep-sea water above the brines. The “isolated island character” of the different brines, that resulted from geological events and contemporary environmental conditions, create selective pressures driving evolutionary processes, and with time, lead to speciation and shape protistan community composition. We conclude that community assembly in DHABs is a mixture of isolated evolution (as evidenced by small changes in V4 primary structure in some taxa) and species sorting (as indicated by the regional absence/presence of individual taxon groups on high levels in taxonomic hierarchy).

## Background

**Figure 1:**
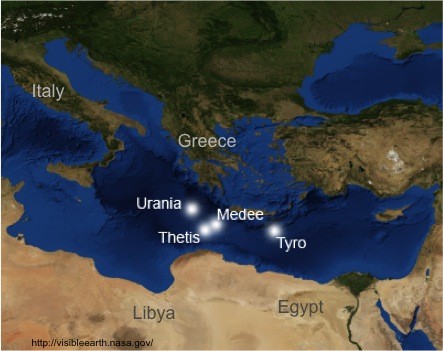
**Map of deep hypersaline anoxic basins (DHABs) sampled in this study (source of satellite image:**http://visibleearth.nasa.gov/**).**

Environmental conditions create selective pressures driving the evolutionary process and creating, over long timescales, a plethora of new species, genera, families and orders [1]. Elucidating mechanisms and environmental factors generating and maintaining biodiversity is one of the major challenges in microbial ecology. We examine evidence for environmental filtering of protistan plankton communities driven by environmental constraints in marine water columns with unique chemistries. As model organisms we targeted the signatures of the ciliated protists (phylum Ciliophora) because this group was found in earlier studies to be a major component of the protistan community, representing 45% of the major taxonomic groups with high alpha diversity [2,3]. Other taxonomic groups with smaller proportions were dinoflagellates, Fungi and Radiolaria (up to 21%, 17% and 11%, respectively) [2]. As study sites, we chose four hypersaline anoxic deep-sea basins (DHABs) located in the Eastern Mediterranean Sea (Figure 1).

DHABs in the Eastern Mediterranean Sea, located at depths of more than 3000 m below sea level, result from the dissolution of outcropping ancient subterranean salt deposits from the Messinian salinity crisis (late Miocene period, > 5 million years ago, [4]). Due to the high densities of the brines (up to 1.23 kg m^-3^, [5]), mixing of these water masses with overlying deep-sea water (average density: 1.03 kg m^-3^) is restricted, resulting in anoxic conditions in these brines. An interface (halocline: depending on the basin, typically 1 to 3 m thick) separates the anoxic brine from the normoxic and normsaline deep-sea water. Due to the dissolution of different strata of the evaporites from the Messinian salinity crisis, the hydrochemistries of the Eastern Mediterranean Sea DHABs differ significantly. For example, while salinity in some basins (Thetis, L’Atalante, Bannock and Tyro) ranges between 321 and 352 g l^-1^ (nearly 10 times higher than average seawater salinity), others exhibit a much lower salinity (such as Urania brine 240 g l^-1^). Potassium ions range between 19 and 300 mmol l^-1^, magnesium ions between 71 and 792 mmol l^-1^ sulfate between 52 and 323 mmol l^-1^, sulfide between 2.1 and 15 mmol l^-1^[5] and methane between 0.4 and 5.6 mmol l^-1^[6]. Because of their unique hydrochemistries and physical separation for thousands of years, the DHABs may serve as island habitats and provide an ideal scenario to test the hypothesis that species sorting of planktonic ciliate communities results from environmental filtering through niche separation.

Molecular diversity surveys of protists, employing domain-specific PCR primers for the amplification of taxonomic marker genes (small subunit ribosomal RNA, SSU rRNA), clone library construction and Sanger sequencing revealed, that ciliates are among the most diverse and abundant plankton taxa thriving in some of the Eastern Mediterranean DHABs [2,3]. Ciliates, through their grazing activities on bacteria, archaea and smaller eukaryotes are central players in the marine microbial loop [7-9] and species composition of ciliates can serve as an indicator of environmental health [10]. They have been used extensively as model organisms to develop and test ideas about microbial biodiversity and biogeography (e.g. [11-17]). One major reason for this is that compared to amoeboid and flagellated organisms, they are morphologically diverse [18,19] and there is a long history of their taxonomic and phylogenetic study (reviewed in [19]). The extensive foundation of knowledge on ciliate species and their inferred relationships facilitates data evaluation and hypothesis testing for studies that aim to explore ciliate biodiversity, evolution and biogeography.

None of the previous taxon samplings of SSU rRNA signatures in initial DHAB protistan diversity surveys reached saturation [2,3], as is generally the case in cloning and Sanger sequencing-based strategies [20-24]. Therefore, it was not possible to observe many patterns of diversity in previous studies of DHABs, nor was it possible to perform comparative statistical analyses of specific taxa in the DHABs. In this study we applied a high-throughput next generation sequencing strategy (pyrosequencing) and a ciliate-specific primer set in order to recover a comprehensive dataset on this target group. The resulting data from deep sequencing enabled us to address basic ecological questions. Our first hypothesis was that the distinct chemistries of the different basins would drive species sorting in planktonic ciliate communities in the brines and interfaces of each basin. If this hypothesis is true, we would expect (i) that interface communities will differ decisively from brine communities (environmental filtering) and (ii) that ciliate communities in interfaces are more similar to each other than in the brines (isolated island character of brine basins). The brines of the different basins are isolated from one another due to the sharp density gradient that exists between these hypersaline basins and overlying Mediterranean seawater. In contrast, exchange may be possible between interface populations in different DHABs since some exchange is possible between seawater and the typically ca. 2 m-thick interfaces (haloclines). Our second hypothesis was that ciliate community composition in the brines and interfaces of these four DHABs, separated by up to 500 km, would not be significantly affected by distance between basins. If this hypothesis is true, we would expect no significant correlation between pairs of samples and geographic distance between the respective sampling sites, therefore, no isolation with distance.

## Results

### Data overview

In total, we obtained between 33,634 (sample Thetis brine) and 80,650 (sample Urania interface) V4-amplicons (Table 1). After quality filtering of the data (including singleton removal), between 32,663 (Thetis brine) and 79,389 (Urania interface) ciliate V4-amplicons remained for further analyses (Table 1). The resulting number of ciliate OTUs called at 95% sequence similarity ranged between 53 (Medee brine) and 551 (Urania brine). After normalization to the smallest dataset (32,663 amplicons) the resulting number of ciliate OTUs ranged between 12 (Medee brine) and 322 (Thetis brine). Sampling saturation curves are presented in Additional file 1: Figure S1. The proportion of rare versus abundant ciliate taxa can be found in Additional file 2: Figure S2. Sequences have been deposited in the GenBank Short Read Archive [SRA061343].

**Table 1:** Summary of ciliate V4 SSU rRNA amplicon data for each sample including the ciliate cluster numbers at a level of 95% cutoff

	**Number of V4-amplicons**		**Number of ciliate clusters**
**Sample**	**Before quality control**	**After quality control**	**Cutoff level 95%**
Tyros IF	36093	35072	245
Tyros B	54885	50713	376
Thetis IF	60173	57937	205
Thetis B	33634	32663	441
Medee IF	42505	40911	152
Medee B	36297	34982	53
Urania IF	80650	79389	237
Urania B	47927	46345	551

### Ciliate diversity in the DHABs

**Figure 2:**
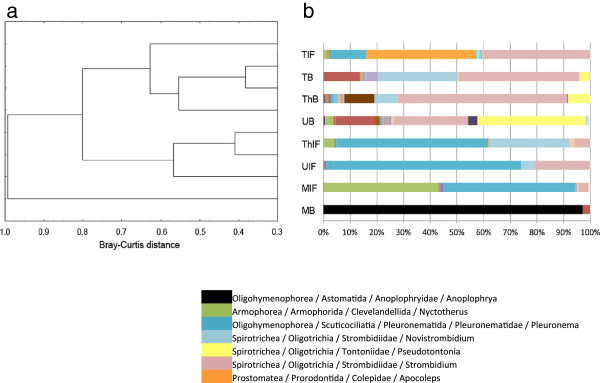
**Hierarchical clustering and taxonomic assignment based on ciliate V4 SSU rRNA-amplicons.** (**a**) Hierarchical clustering (Bray-Curtis distance) of sampling sites based on ciliate community profiles in four DHAB halocline interfaces (IF) and brines (B). (**b**) Taxonomic assignment of ciliate V4 SSU rRNA-amplicons. In total, all amplicons could be assigned to 102 different ciliate genera (closest BLAST match in GenBank nr database) and one unclassified. In the legend of the figure we only show the taxa that are represented by at least 20% of all amplicons in at least one of the eight samples. For further details on taxonomic assignments we refer to Additional file 3: Table S1. M = Medee, T = Tyro, Th = Thetis, U = Urania.

Hierarchical clustering of sampling sites based on Bray-Curtis distance (Figure 2a) identified two clusters, one of which unites the brine ciliate communities of the basins Tyro (TB), Thetis (ThB) and Urania (UB), and, distantly related to these brine communities, the ciliate community from Tyro interface (TIF). The parametric estimator ACE predicted highest ciliate richness in TIF (58.0, Table 2). Tyro brine, Thetis brine and Urania brine shared most ciliate amplicons. The Shannon index (Table 2) indicated the highest ciliate diversity in these three samples (Thetis brine 1.37; Tyro brine 1.48; Urania brine 1.73). The second cluster included the interface ciliate communities from Thetis (ThIF), Urania (UIF) and Medee (MIF). The Medee brine (MB) ciliate community was distinct from all other ciliate communities analyzed in this study. The Shannon diversity index of Medee brine was the lowest of all communities analyzed (0.14, Table 2), and also richness estimates were distinctively lower than for all other samples (ACE = 16.9, Table 2).

**Table 2:** Alpha diversity indices (data normalized to 32,663 sequences in each sample) of ciliate communities in DHAB interfaces and brines

	**Shannon index**	**ACE**
Tyro interface	1.285 ± 0.002	58.0 ± 3.3
Tyro brine	1.477 ± 0.004	44.6 ± 3.5
Thetis interface	1.139 ± 0.004	42.4 ± 3.4
Thetis brine	1.370	44.3
Medee interface	1.067 ± 0.003	42.9 ± 2.0
Medee brine	0.142 ± 0.001	16.9 ± 1.2
Urania interface	0.895 ± 0.004	33.9 ± 6.5
Urania brine	1.730 ± 0.004	47.5 ± 3.0

### Putative taxonomy of ciliate amplicons

The V4-amplicons analyzed in this study were related to a total of 102 identified ciliate genera and one unclassified ciliate taxon (Additional file 3: Table S1). The unique character of the Medee brine ciliate community can be inferred from Figure 2b, which displays the taxonomy assigned to the ciliate amplicons obtained from each sampling site. Medee brine was dominated by amplicons (n = 33,961; 97% of all amplicons), which were all related to the genus *Anoplophrya* (Astomatida) as closest BLAST match in NCBIs GenBank nr database. The sequence similarities of these amplicons to *Anoplophrya* ranged between 80 and 89% (Additional file 4: Table S2). The remaining 1021 ciliate amplicons from Medee brine were related to a few other taxon groups belonging predominantly to the Peniculida (2.7%), other Astomatida (0.06%), and Pleuronematida (0.03%). Thetis brine and Tyro brine had a relatively similar ciliate community composition, both of which were dominated by amplicons that have *Strombidium* as the closest BLAST match in the GenBank nucleotide database (64% and 45%, of all amplicons, respectively). Other abundant taxon groups shared by these two samples were *Novistrombidium* (30% in Tyro brine and 9% in Thetis brine), and *Pseudotontonia* (4% in Tyro brine and 8% in Thetis brine). While *Laboea* accounted for 11% of all amplicons in Thetis brine, this taxon group was absent in Tyro brine. A tintinnid ciliate taxon related to *Salpingella* as closest database relative occured exclusively in Tyro brine (4% of all amplicons), but not in Thetis (Additional file 3: Table S1). The ciliate community composition in Urania brine was dissimilar to the brines in Tyro and Thetis basins. One striking quantitative difference was the high proportion of *Pseudotontonia*-related amplicons (40%) in Urania brine. However, while most of the relatively abundant taxon-groups were shared between these three brine samples (but in different quantities), most qualitative differences between Tyro, Thetis and Urania brines were attributed to taxon groups with lower abundances. Medee brine was distinct in its ciliate composition from other brines.

Tyro interface stood out from the other interface samples. The most significant difference was the occurrence of 14,337 amplicons (41%), with *Apocoleps* (Prorodontida) as the best BLAST match. The proportion of amplicons in Thetis, Urania and Medee interfaces related to this taxon was less than 0.5%. Also the proportion of *Strombidium*-like amplicons in Tyro interface (40%) was decisively higher compared to the other interfaces (4-21%). Thetis interface and Urania interface had a very similar taxon composition, dominated by amplicons most closely related to *Pleuronema* (Pleuronematida) (70% in UIF and 57% in ThIF). This taxon was also highly represented in Medee interface (49%). The second most abundant taxon group in Medee interface were clevelandellids, represented with 43%. This taxon was underrepresented in the interfaces of other basins (0.02% in UIF – 4% in ThIF).

Four taxa occured in all eight samples analyzed (closest BLAST matches: *Pleuronema*, *Strombidium, Omegastrombidium, Apocoleps*). Four taxa were exclusive to all interfaces (*Palgiopyliella*, *Cyclidium*, *Schizocalpytra*, *Isochonida*). Interestingly, not a single taxon occured exclusively in all brines simultaneously. However, 28 taxon groups were absent from interfaces but present in at least one of the brines. The same number of taxon groups was absent from all brines but occured in at least one of the interfaces. The majority of taxon groups had abundances accounting for less than 5% of all amplicons obtained within a sample.

### Relating community patterns to environmental variables

**Figure 3:**
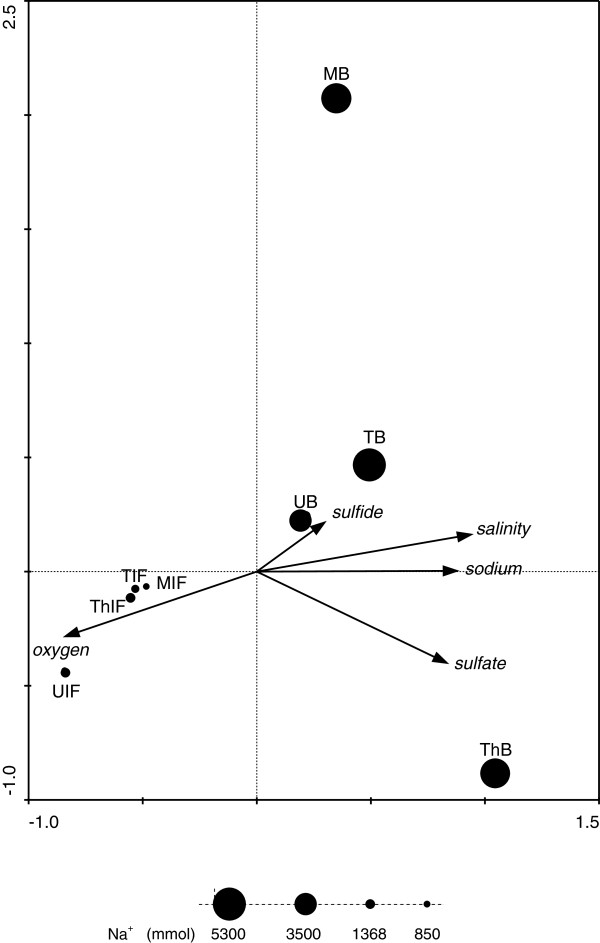
**Canonical correspondence analysis (CCA) of ciliate V4 SSU rRNA- amplicon profiles for brines (B) and halocline interfaces (IF) of the different sampling sites.** This CCA depicts the best model in our CCAs, explaining 71.4% of the total variation within the community profiles with the first two axes accounting for 41% of community composition variance. The first two canonical axes (most important synthetic gradients) explained 51% of the variation of the species-environment relation. Sodium concentration is significantly (positively) correlated with the second axis (p = 0.003). Bubble sizes correspond to Na^+^ concentration in each sample. M = Medee, T = Tyro, Th = Thetis, U = Urania.

We used a Canonical Correspondence Analysis (CCA) to extract environmental gradients from the observed partitioning of ciliate amplicon diversity. In the resulting ordination diagram (Figure 3), environmental variables with arrows close to the canonical axes may explain a large proportion of the variation accounted for by this axis. The longer the arrow, the more variation may be explained by this factor. The best model in our CCA explained 71.4% of the total variation within the ciliate amplicon profiles with the first two axes (= two best synthetic gradients) accounting for 41.4% and the first two canonical axes explaining 50.8% of the variation of the species-environment relation. Eigenvalues of axis 1 and axis 2 were similar (0.388 and 0.349, respectively). While all interface samples (IF) were at the left part (negative scale) of axis 2, all brine samples were distributed along its positive scale of values. Even though only sodium concentration was significantly correlated with the second axis (p < 0.01) also oxygen concentration and salinity described the differential habitat preferences of the communities distributed along the second canonical axis. Thus, these factors can be identified as main explainable environmental selection factors for interface and brine ciliate community composition (niche separation).

The ciliate communities in the DHAB interfaces showed only small variation along the first axis, while brine samples spread across a wider range of this first axis, with Medee brine and Thetis brine defining the longest distance. None of the CCAs conducted found a meaningful correlation of this axis with any environmental variable that we have measured and tested explaining this first axis. However, it must be a factor that only separates niches for the brine communities, but not for interface communities.

### Distance effect on DHAB ciliate community profiles

**Figure 4:**
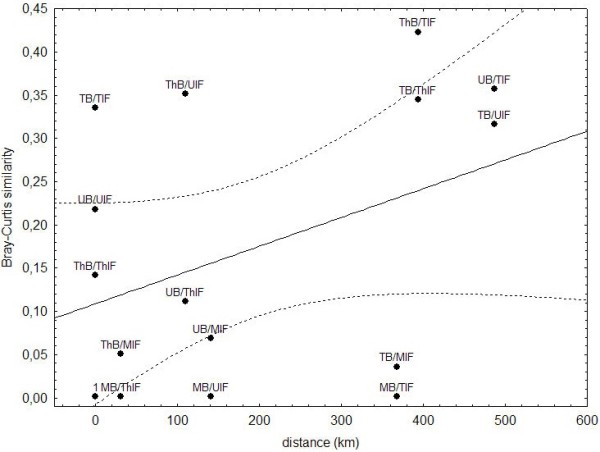
**Regression analyses of Bray-Curtis similarity between pairs of samples and the geographic distance between the respective sampling sites.** Only little of the overall variability in protistan community similarities was accounted for by the regression model (R^2^ = 0.16). A Pearson-rank correlation between distance and community similarity is insignificant (p = 0.13). Dotted lines represent 95% confidence intervals of the regression model.

Distance dependence was low (Figure 4), and very little of the overall variability in ciliate community similarity was accounted for by the regression model (R^2^ = 0.16). A correlation between distance and community similarity was insignificant (p = 0.13, Pearson-rank correlation). A permutation Mantel test between the geographic distance and the Bray Curtis distance showed also a non-significant correlation (p = 0.178).

### Fluorescent in situ hybridization and scanning electron microscopy

**Figure 5:**
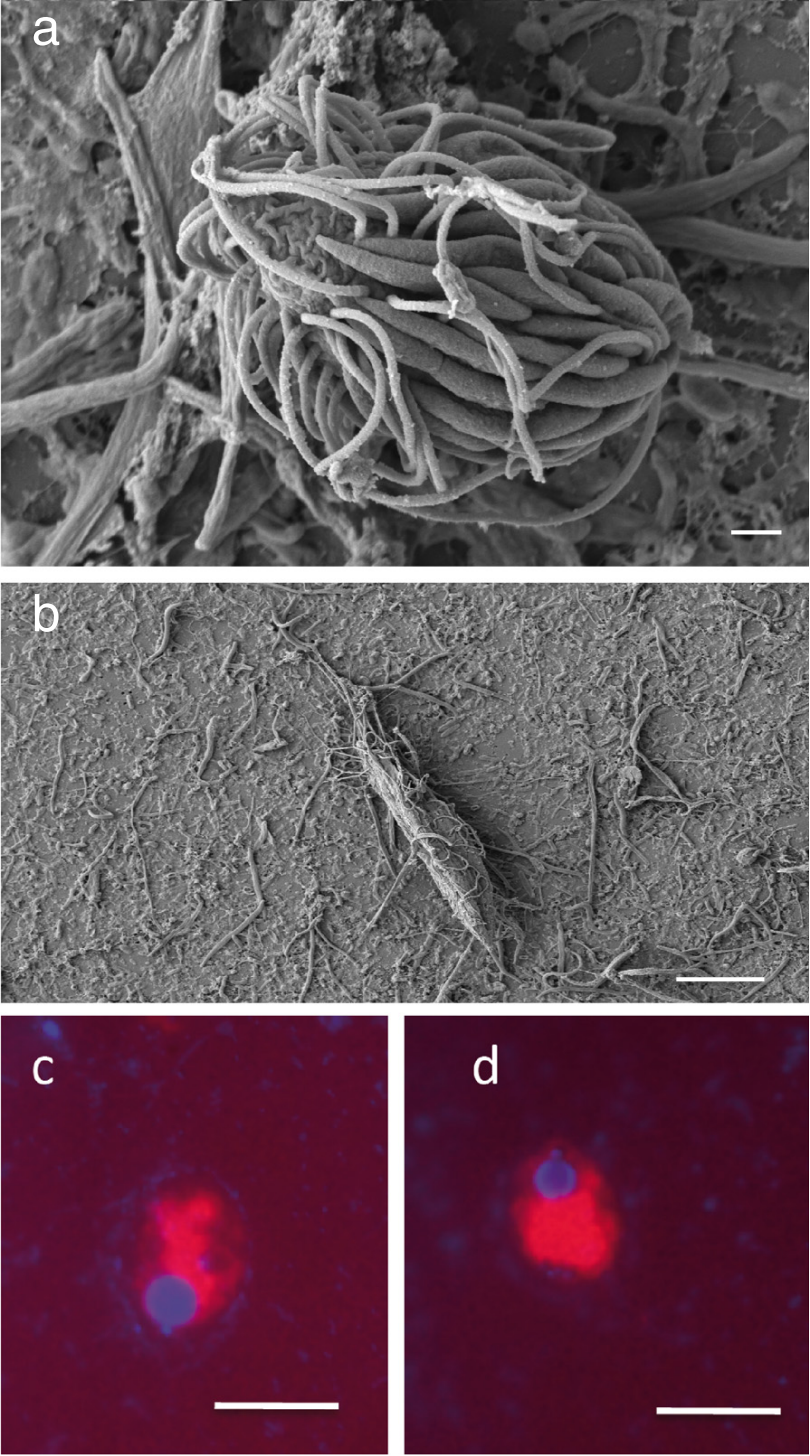
**Scanning electron microscopy (SEM) and fluorescent in situ hybridization (FISH) images of ciliates. a)** SEM of scuticociliate morphotype from Urania interface, EHT = 3 kV, Signal A = SE2, WD = 9.7 mm, Width = 15.99 μm, scale 1 μm, **b**) fusiform ciliate from Urania interface, WD = 10 mm, Width = 91.74 μm, scale 10 μm. a-b: with MBL, Biological Discovery in Woods Hole. **c-d**) FISH images of ciliate morphotypes from Urania brine (general eukaryotic probe EUK1209). Scale in c-d 5 μm.

Scanning electron microscopy performed on samples collected from Urania halocline revealed abundant ciliates (95% scuticociliate morphotype) present at a concentration of 9.7 (+/− 0.2) × 10^4^ cells L^-1^), all of which hosted bacterial epibionts approximately 2–2.5 μm long that ([25]; Figure 5). These results supported the decision to focus on ciliates only in this work. SEM was not performed on brine or interface samples from the other basins, however FISH hybridizations with the general eukaryotic probe Euk1209 confirmed the presence of ciliates (with visible macro- and micro-nuclei) in Urania brine.

## Discussion

Deep hypersaline anoxic basins (DHABs) in the Eastern Mediterranean Sea are ideally suited for testing the effect of historical contingencies on the evolution of protist communities. The distance between individual basins is variable, and each basin is characterized by hydrochemical gradients (interfaces to brines), and slightly different origins, leading to differences in physicochemical factors of the brines and interfaces in each of the different basins. Due to the steep density gradients along the interfaces of these basins, there is little connectivity between basin brines and overlying seawater, and therefore, between basin brines. First insights into the ciliate communities in the mesopelagic realm above the brine basins came from a Sanger sequencing-based approach [3]. Because of the relatively small amount of data (four ciliate OTUs in the mesopelagic reference and 10 in the brine) it is not a reliable dataset for comparison to the high throughput sequencing data from this study. However, the data from that preliminary study did indicate a significant community shift between the water column and the basin brines. We assessed ciliate community structures in the interfaces and brines of several basins in order to determine the degree to which these environmental barriers and basin chemistries influenced the ciliate plankton.

The proportion of rare versus abundant ciliate taxa in this study (Additional file 2: Figure S2) is comparable to previous findings reporting protistan communities with few abundant and many rare taxa [16,24]. Several molecular diversity surveys over different spatial scales ranging from centimeters to tens of thousands of kilometers have supported distance-decay relationships (effect of distance on spatial interactions) for microbial organisms, including bacteria (e.g. [26,27]), archaea (e.g. [28]), fungi (e.g. [29]) and also protists (e.g. [30-32]). Even organisms with large population sizes and the potential to spread globally using spores, which were assumed to be cosmopolitan [13,33], show significant non-random spatial distribution patterns [34]. However, in our study of ciliate communities in these DHABs, a similar distance-decay relationship was not observed (insignificant correlation between Bray-Curtis and geographic distances in Pearson correlation and Mantel test). A potential explanation could be that the small number of compared locations may have masked true patterns. Alternatively, the presence of a metacommunity [35] within the Mediterranean Sea could cause the absence of a significant heterogeneous distribution [36,37]. In limnic systems geographic distance has been found to influence asymmetric latitudinal genus richness patterns between 42° S and the pole [32]. However, this seems to be a fundamental difference between marine and “terrestrial” (land-locked) systems. Furthermore, on a global scale, historical factors were significantly more responsible for the geographic patterns in community composition of diatoms than environmental conditions [32]. In other marine studies ciliates showed variations in taxonomic composition between closely related samples, which were explained by environmental factors rather than distance [38]. Similarly, in our study geographic distance could not explain the variations observed between the ciliate communities. Instead, hydrochemistry explained some of the variation in observed ciliate community patterns, and there was a strong separation of halocline interface and brine communities (Figure 3). The DHAB interfaces are characterized by extremely steep physicochemical gradients on a small spatial scale typically less than a couple of meters (for example, only 70 cm in Medee, [39]). The concentrations of salt and oxygen are the most prominent environmental factors that change dramatically along the interfaces into the brines. In a recent metadata-analysis of environmental sequence data, these two factors were identified as strong selection factors for ciliates [40]. Also for bacterial communities, salt concentration emerged as the strongest factor influencing global distribution [41]. Likewise, the bacterioplankton community composition in coastal Antarctic lakes was weakly related with geographical distance, but strongly correlated with salinity [42]. Accordingly, Logares *et al.* assumed that a long-term salinity change ‘promoted the diversification of bacterioplankton communities by modifying the composition of ancestral communities and by allowing the establishment of new taxa’ [42]. Salinity shifts characterize a boundary which is one of the most difficult barriers to cross for organisms in all three domains of life [43]. While mechanisms to cope with high salt concentrations are relatively well studied in prokaryotes, they are still largely unknown in protists (with the exception of the model algae *Dunaliella salina*[44]). While there is evidence that many protists have narrow ranges of salt tolerance [45,46], some taxa are known to occur under a wide range of salinities, from freshwater to hypersaline [47]. One example is the ciliate *Cyclidium glaucoma*[48], which may explain the occurrence of some of the same phylotypes in haloclines and brines of specific DHABs. Other examples are likely to exist.

In contrast, adaptations to anoxia in ciliates are well known. Ciliates are one of the most successful eukaryotic taxon groups in hypoxic and anoxic habitats. In their long evolutionary history, they have acquired several strategies that allow for an anaerobic lifestyle, including hydrogenosomes [49,50], anaerobic mitochondria [51], and/or symbiotic networks [52,53]. The high taxonomic diversity of anaerobe ciliates includes taxa such as *Nyctotherus, Loxodes, Pleuronema, Strombidium, Trimyema*, *Cyclidium* and *Metopus*, some of which were also detected in our genetic diversity survey. Electron microscopy and fluorescence *in situ* hybridization assays provide unbiased evidence that the genetic signatures we detected in our rRNA-targeted gene survey can be assigned to ciliates living in the DHABs rather than reflecting ancient nucleic acids. (Figure 5, [25,54]). Taking advantage of phylotypes that we detected exclusively in specific habitats and phylotypes that can be found in several habitats with distinct hydrochemical characteristics, we may assume that the latter have a character of more generalist taxa compared to the more locally restricted phylotypes. The total number of observed taxon groups is 102 distributed over eight different datasets (samples or habitats) (Additional file 1: Figure S1). In those eight samples there are 13 generalist taxonomic groups that appeared simultaneously in at least six of the datasets. Only four taxonomic groups appeared in all of the eight datasets. Specialists, i.e. taxa that are restricted to a single unique habitat account for 34 different taxonomic groups. This results in a specialist/generalist ratio of 8.5 to 1, indicating a high specialization of taxa in the habitats under study*.* However, there is a limitation to infer the autecology of specific evolutionary lineages based on sequence data and microscopy evidence [25]. We do not make any attempt to explain the presence or absence of specific phylotypes in individual samples, and we instead focus only on community level ciliate diversity.

Hydrochemical gradients have been identified as environmental barriers in previous protistan diversity surveys including the Cariaco basin [55], Framvaren Fjord [56], Mariager Fjord [57], Baltic Sea [58] and the Black Sea [59] and also stratified lakes [60]. Therefore, the clear distinction of halocline ciliate communities from brine communities is not an unexpected result. However, it is surprising that the environmental variables we measured had a minor contribution to differences among the individual brine ciliate communities. In the CCA analyses (Figure 3) the different brine communities were spread out along the y-axis. This axis, however, does not represent an environmental gradient. This is surprising, considering that different types of salts may have different physiological effects [61] and therefore, should require different adaptation strategies in halophiles. Basically, we can assume two scenarios: first, for isolated evolution as described in [62], the scenario starts with a seed taxon. After physical separation of the original habitat into two habitats neutral mutations are changing the seed taxon in these habitats independently. These neutral mutations are of minor nature considering the time scale of the basins’ geological histories. From this event we would expect similar taxon groups with only minor genetic changes in both habitats. As mentioned above, each eighth taxon recorded in our study (Additional file 3: Table S1) falls into this category. In the second scenario (environmental filtering) we have the same ‘seed bank’ community for different basins. Through environmental filtering (different hydrochemistries of the basins) some taxa may go extinct, others have the genomic potential to adapt to some specific hydrochemistries, while others are genomically equipped for adaptation to other environmental conditions. In this case we would find taxa differing on higher taxonomic (genetic) hierarchies. This is the case for 34 of 102 detected taxon groups (Additional file 3: Table S1). We cannot rule out all environmental factors from causing differences between the ciliate communities because we did not measure all possible environmental factors, but only the hydrogeochemical factors that account for the most pronounced and obvious differences. This suggests that (1) other hydrochemical variables we did not measure are leading to this separation, or (2) that biotic interactions may explain some of the differences between brine ciliate communities. Even though interactions of top-down and bottom-up factors in shaping community structures of aquatic microbes are still poorly understood [63] some well known biotic interactions could be considered. Such biotic interactions may be, for example, parasitic relationships between organisms like amoeboid parasitic forms that can shape the composition of cyanobacterial species in lakes (Rohrlack et al., unpublished data). Furthermore, symbiotic relationships include ciliates, associated with epibiontic prokaryotes, were found to be the dominant eukaryotic morphotypes in the haloclines of DHABs in the Mediterranean Sea [25]. Biotic interaction between protists and viruses are also known and have been shown [64]. Viruses specifically infect protists, e.g. the *Coccolithovirus* and it’s host, the calicifying haptophyte *Emiliania huxleyi*[65]. Additionally, viruses can also have an an indirect influence on protists by infecting the bacteria on which the protistan grazers feed or protistan grazers can even feed directly on viruses even though the carbon transfer to the higher trophic level is of minor importance [66]. Furthermore, different bacterioplankton communities can produce a bottom-up control on grazing protists. Namely, the growth efficiency of protists can relate strongly to the available bacterial prey [63,67]. This is highly likely because differences in bacterial community composition in DHABs have been shown before [68,69]. That leads to the assumption that different bacterial communities support different phagotrophic protists that show strong preferences for particular prey species [63,67,70,71] or morphotypes [72,73]. Other possible explanations are founder effects, which describe a genetic deviation of an isolated population or founder population (on an island for example) compared to the original population based on a low number of alleles within the founders individuals [74], random effects or genetic drift is the change in the frequency of a gene in a population due to random sampling [75] and random extinctions that describe when a gene causes its carriers to have a deviating fitness from unity, its frequency will be determined by selection [76] in different basins. For protists in particular there is no literature available on this topic to our knowledge. At last, the Monoplization Hypothesis by De Meester et al. [77] could be relevant to protist biogeography stating that a fast population growth and local adaptation and colonization of a new habitat result in the monopolization of resources, which yields a strong priority effect. The effect is even enhanced when a locally adapted population can provide a ‘large resting propagule bank’ as a strong buffer against new genotypes invading. This holds true especially for species that reproduce asexually and form resting stages.

Even though mass effect and dispersal [78] cannot be ruled out, these are unlikely alternatives to explain the observed community patterns. The habitats of the water column above the DHABs represent a potential source habitat with ‘high quality’. In comparison, the narrow interphase and the brine show ‘low quality’ conditions because these habitats harbor high gradients of change, anoxia, high salt concentration up to saturation and therefore require a high degree of physiological adaptation for microbial colonization. Chances for highly specialized organisms to cross environmental barriers outside their habitat and to disperse beyond their specific habitat are very low. Evidence for this hypothesis comes, for example, from the very rare and infrequent dispersal of protists between marine and freshwater habitats [43,79]. One possibility may be the dispersal of spores and/or cysts (resting stages), however, our knowledge about the number of ciliates that can form such resting stages in nature is very limited [80]. Furthermore, physical mechanisms of transport for resting stages between different basins are difficult to imagine, considering the lack of fluid flow, high density, and lack of animal vectors in the brines. In contrast, this scenario may be more plausible for cysts/spores in halocline/interphase habitats. Physical transport of resting stages between haloclines at different basin sites could explain the observed similarities in ciliate interphase communities (Figure 3).

The deep basins in the eastern Mediterranean Sea may have recruited their protistan seed communities from Atlantic Sea water during the Zenclean Flood (~5.3 mya), when the Strait of Gibraltar opened permanently and refilled the mostly dried out Mediterranean Sea [81]. Subsequently, due to the dissolution of evaporites and the rise of anoxia in deep basins the water masses became physically separated from each other. Anoxia and hydrochemistry likely exerted an increased pressure on the original protistan communities. Species sorting may have been driven through environmental filtering [37,42,62,82]. This is a predictable and fundamental process of community assembly [83], that allows only those taxa with the genomic and physiological potential to cope with each specific set of environmental conditions. This has been evidenced for recent ciliate communities [40]. The normsaline and normoxic deep-sea water separating the different hypersaline anoxic basins from each other then became an environmental barrier for most protists (with the exception of cyst-forming taxa), with the consequence that genetic exchange among the different brines was no longer likely. Changes in the SSU are presumably neutral, therefore, these changes would be due to random mutations. However, it is reasonable to assume that changes in the SSU rDNA are occurring in congruency with whole genome changes and not independent of evolutionary genome processes. Evolution over geological time may have resulted in significantly different ciliate communities in the brines. Divergence of species occurring in isolation through adaptive shifts that occurs in common seed species populations has been demonstrated for a number of taxa, including several macro- and microinvertebrates using molecular as well as taxonomic studies [84-87]. Based on our data, it is not unreasonable to assume that protists are also subjected to such evolutionary processes. Our study strongly suggests that evolutionary time scales combined with physical and hydrochemical isolation can explain, in part, the observed evolutionary differences in the ciliate communities in the different DHABs studied here.

## Conclusions

The data presented here suggests that ancient isolated habitats, like deep-sea brine lakes, which also occur in the Red Sea [88] and the Gulf of Mexico [89] or habitats like aquifers and cave systems will represent ‘hot spots’ for the discovery of as-of-yet unstudied, possibly highly divergent, and endemic microbial eukaryotes.

### Financial competing interests

In the past five years we did not receive reimbursements, fees, funding, or salary from an organization that may in any way gain or lose financially from the publication of this manuscript, either now or in the future. We do not hold any stocks or shares in an organization that may in any way gain or lose financially from the publication of this manuscript, either now or in the future. We neither hold nor apply for any patents relating to the content of the manuscript. We did not receive reimbursements, fees, funding, or salary from an organization that holds or has applied for patents relating to the content of the manuscript. We, the authors, do not have any other financial competing interests.

### Non-financial competing interests

There are no non-financial competing interests (political, personal, religious, ideological, academic, intellectual, commercial or any other) to declare in relation to this manuscript.

## Methods

### Sampling

The seawater-brine interfaces (haloclines) of the DHABs Tyro, Thetis, and Medee in the Mediterranean Sea were sampled on the cruise aboard the *R/V Urania* in 2009. Samples from the DHAB Urania were collected in 2009 on the *R/V Oceanus*. Sampling sites are depicted in Figure 1 and coordinates with environmental data for each DHAB halocline and brine are provided in Table 3. The positions of the interfaces were determined using a SBE911*plus* CTD (Sea-Bird Electronics, Bellevue, WA, USA) equipped with an SBE43 oxygen sensor (Sea-Bird Electronics, USA). Samples were collected from the interface and brine of each basin using a rosette equipped with 12-L Niskin bottles. The salinity gradient from the top to the bottom of individual Niskin bottles was confirmed on board the ship using a WTW portable sensor for conductivity, pH and temperature (WTW, Weinheim, Germany). Water samples were collected from Niskin bottles into 50-L Nalgene bottles flushed with argon gas and 6–10 L water were filtered immediately onto Durapore membranes (47 mm; 0.65 μm; Millipore, USA) under gentle vacuum (flow rate: ca. 50 ml/min) and under argon in the case of anoxic samples [2], followed by storage in RNA*later* (Ambion, Applied Biosystems, USA). According to Ambion’s RNA*later* manual, the filters were stored at 4°C for 24 hours prior to freezing at −20°C until RNA extraction. RNA was used to ensure that samples were not contaminated by settling DNA from above the investigated layers.

**Table 3:** Coordinates, sampling depths and physico-chemical data of the brines (B) and halocline interfaces (IF) of the different DHABs under study

	**Coordinates (Long, Lat)**	**Depth (m)**	**Salinity**^**a**^**(PSU)**	**Conductivity**^**a**^**(S/m)**	**Oxygen**^**a**^**(ml/l)**	**Na**^**+**^**(mmol)**	**Mg**^**2+**^**(mmol)**	**SO**_**4**_^**2-**^**(mmol)**	**HS**^**-**^**(mmol)**
MIF	22.312124 E, 34.19468 N	2924	70	7.7	0.5	847	161	41	n.a.
TIF	26.21962 E, 33.524236 N	3327	67	7.8	0.5	1111	15	11	0.07
ThIF	22.084368 E, 34.401134 N	3259	80	8.2	0.68	1368	174	76	0.11
UIF	21.283252 E, 35.13528 N	3468	63	7.8	1.22	876	79	42	0.66
MB	22.312124 E, 34.19468 N	2950	320	16.7	0	4818	792	201	2.9
TB	26.21962 E, 33.524236 N	3448	321	16.7	0	5300^b^	71^b^	53^b^	2.1^b^
ThB	22.084368 E, 34.401134 N	3380	348	16.7	0	4760^b^	604^b^	265^b^	2.1^b^
UB	21.283252 E, 35.13528 N	3493	240	15.6	0	3505^b^	315^b^	107^b^	15

*M* Medee, *T* Tyro, *Th* Thetis, *U* Urania. Data are from the literature and from this study (measured as described in [5]). *n.a.* not available.

^a^from [54]; ^b^from [5].

### Environmental RNA Isolation, transcription and PCR amplification of ciliate SSU rRNAs

The method for the extraction and reverse transcription of environmental RNA (envRNA) from protistan plankton collected on membranes has been described in detail previously [2]. In short, total RNA was extracted using Qiagen’s AllPrep DNA/RNA Mini kit (Qiagen, Germany) according to the manufacturer’s instructions following a chemo-mechanical cell disruption by bead-beating (45 s, 30 Hz). Residual DNA was removed by DNase I (Qiagen) digestion. We conducted a PCR with the digested RNA to exclude the possibility of residual DNA in downstream applications (PCR protocol see below). The concentration of extracted and purified RNA was determined spectrophotometrically using a Nanodrop ND-1000 UV–vis spectrophotometer (Nanodrop Technologies, Wilmington, DE, USA). The integrity of the RNA was checked with an RNA 6000 picoassay on an Agilent 2100 Bioanalyzer (Agilent Technologies, Germany). To minimize extraction bias, total RNA from three individual filters (each representing 6-10 L water) per depth and sampling site were extracted. Total RNA was then reverse transcribed into cDNA using Qiagen’s QuantiTect Reverse Transcription kit and with random primers provided with the kit according to the manufacturer’s instructions. After transcription of each individual sample, the three replicate transcribed products of each depth/sampling site were pooled and subjected to SSU cDNA amplification. First, amplification with a ciliate specific primer set (Table 4) was performed to filter specifically the ciliate SSU rRNA from the env cDNA. The PCR reaction included 50–100 ng of template cDNA in a 50 μl-reaction, 1 U of Phusion High-Fidelity DNA polymerase (Finzymes), 1× Phusion HF Buffer, 200 μM of each deoxynucleotide triphosphate, and 0.5 μM of each oligonucleotide primer. The PCR protocol amplifying ca. 700 bp-long gene fragments consisted of an initial denaturation (30 s at 98°C) followed by 30 identical amplification cycles (denaturation at 98°C for 10 s, annealing at 59°C for 10 s and extension at 72°C for 30 s), and a final extension at 72°C for 10 min. Subsequently, the purified (Qiagen’s MiniElute kit) PCR products from the first reaction were subjected to a second PCR, which employed eukaryote-specific primers for the amplification of the hypervariable V4 region ([16]; Table 4). The PCR protocol started with 10 identical amplification cycles at an annealing temperature of 57°C where only the forward primer would operate, followed by 25 cycles with a primer annealing at 49°C where both forward and reverse V4 primers would amplify [16]. The resulting PCR amplicons (ca. 480 bp) were excised from the gel using Qiagen’s Gel extraction kit. Gel extraction eliminates unspecific shorter fragments, invisible on a gel, in the final amplicon library. The integrity and length of purified amplicons was determined with a DNA 500 LabChip on an Agilent 2100 Bioanalyzer.

**Table 4:** Primer sets used in this study for the specific amplification of ciliate V4-SSU rRNA fragments using a two-step (nested) PCR reaction

			**Primer**	**Primer sequences**	**Reference**
1. Reaction: PCR ciliate specific primers					
			Cil F	5′-TGGTAGTGTATTGGACWACCA-3′	[105]
			Cil R1	5′-TCTGATCGTCTTTGATCCCTT-3′	[105]
			Cil R2	5′-TCTRATCGTCTTTGATCCCCTA-3′	[105]
			Cil R3	5′-TCTGATTGTCTTTGATCCCCT-3′	[105]
2. Reaction: PCR hyper variable V4-region					
**Linker sequences**	**Key**	**MID**	**Primer**	**Primer sequences**	**Reference**
5′-CGTATCGCCTCCCTCGCGCCA	TCAG	MID	TAReuk454FWD1	5′-CCAGCASCYGCGGTAATTCC-3′	[16]
5′-CGTATCGCCTCCCTCGCGCCA	TCAG	MID	TAReukREV3	5′-ACTTTCGTTCTTGATYRA-3′	[16]

### Pyrosequencing and sequence data processing

The DNA sequencing of the V4-amplicons was conducted by Engencore (University of South Carolina, USA) using Roche’s Titanium chemistry. One half plate was sequenced with the eight different samples with individual MIDs. The number of amplicons obtained after sequencing ranged between 33,634 (Thetis brine) and 80,650 (Urania interface) sequences.

For sequence data quality control and processing, we used the program JAguc [90]. All tags that met any of the following conditions were considered as “low quality” and removed from further analyses: sequences <200 nucleotides, sequences containing an inaccurate calibration key, incomplete or erroneous forward and reverse primer sequences, presence of an ambiguity code. Sequences were then clustered. A cluster included sequences that shared at least 95% similarity in their primary structures. This conservative cluster threshold was chosen, because it accounts for sequencing errors and for intraspecific variability in the hypervariable SSU rDNA V4 region of ciliates [91,92]. Single singletons (unique amplicons after 95% clustering that occurred exclusively in only one of the eight samples) were removed from downstream analyses as they are most likely erroneous sequencing products [91,93].

### Taxonomic assignment

We assigned taxonomy to each amplicon by conducting BLASTn searches implemented in JAguc (using parameters -m 7 -r 5 -q −4 -G 8 -E 6 -b 50) of each unique tag against a local installation of NCBI’s nucleotide database (nr/nt, release 187). Only unique tags with a best BLAST hit of at least 80% sequence similarity were assigned to a taxonomic category. The remaining tags were assigned to an artificial category “others”. This information was stored in JAguc’s database. We only assigned taxonomic labels to the genus level, because taxon assignments on lower taxonomic levels become inaccurate and biased due to the relatively limited sequence information provided in short amplicons [92]. Taxonomy of ciliates follows the compendium “The Ciliated Protozoa” by D. Lynn [19].

### Statistical analyses of ciliate amplicon profiles

To assess the ciliate diversity within a particular sample (alpha-diversity, [94]), we normalized the data (to the smallest number of sequences: 32,663 sequences were picked randomly 10,000 times in each of the samples with the software R [95]). We used the Shannon index (combining richness and relative abundance; [96]) and the non-parametric richness estimator ACE [97] as calculated with R [95]. The partitioning of diversity between communities (beta-diversity) was calculated with the Bray-Curtis index, using the software EstimateS v.8 [98], and then translated into distance matrixes (1 minus Bray-Curtis index value) for UPGMA cluster analyses. Bray-Curtis similarity index is a modified version of the Sørensen index, which considers abundance distribution (also known as the Sørensen abundance Index or the quantitative Sørensen index [99,100].

To assess an effect of distance on community similarities, Jaccard and Chao-Sørensen indices were plotted against distance data among individual sample sites in a Pearson-rank correlation using the Statistica software package. A Student’s t-test for paired samples was used for significance testing. A Mantel test between the geographic distance and the Bray Curtis distance matrices was conducted to evaluate the significance of the correlation coefficient between geographic and genetic distance. The Mantel test was conducted using the software add-in for Microsoft Excel XLSTAT (http://www.xlstat.com) with 10000 permutations. Geographical distances were calculated via the subtraction of different depths on a single geographical position, which resulted in the altitude difference within the same basin. For the calculation of the 2-dimensional great-circle distance between two points on a sphere from their longitudes and latitudes (same depth) the haversine formula [101] was implemented in the script as provided by Chris Veness (2002–2011) at http://www.movable-type.co.uk/scripts/latlong.html.

A canonical correspondence analysis (CCA) of quantitative amplicon profiles was conducted to describe the relationships between ciliate community composition patterns and underlying environmental gradients, which shape these diversity patterns. Data were log-transformed [102] and unconstrained permutations (n = 499) were run under a reduced model. Monte Carlo significance tests of first ordination axes and of all canonical axes together were performed. Initially, all available environmental variables (see above) were included in the model. In order to develop a robust model explaining as much variance as possible while avoiding multi-colinearity, individual variables were removed in a step-wise manner. We used the Canoco software (Microcomputer Power, Ithaca, NY, USA) for the ordination analysis.

### Scanning electron microscopy (SEM) preparation and enumeration of ciliates

We used SEM to visualize ciliate morphotypes and to amend the molecular diversity survey with imaging analyses. We followed the method for SEM described in [25,103]. In short, fixed samples were filtered onto 0.4-μm polycarbonate Transwell membrane filters (Corning, USA) and washed with 1X PBS (pH 7.4) that were taken through a dehydration series and fixed with 100% hexamethyldisilizane (Electron Microscopy Sciences, Hatfield, Pennsylvania) before air-drying. Transwell filters were not exposed to air at any point during the protocol, until the final step to prevent collapse of fixed protists. Filters were attached to a carbon adhesive tab and mounted on a SEM specimen holder. Mounted specimens were then sputter coated with 10–15 nm of gold and palladium (60:40) using a Tousimis Samsputter 2A and visualized with a Hitachi S4800 scanning electron microscope. A minimum of 50 microscopic fields (0.5 × 1.0 mm) were observed to count ciliates in each of the samples, and ciliates were counted on at least three different filters.

### Fluorescence *in situ* hybridization

In order to evaluate the relative abundance of ciliates as part of the protistan assemblages we used fluorescence *in situ* hybridization with a specific oligonucleotide probe. FISH followed the protocol of [103]. In short, 100–150 ml of paraformaldehyde-fixed (2% final concentration) seawater was filtered onto 0.65 μm filters and frozen at −20°C. Filters were thawed, and cut into small triangles before the hybridization step and ~20 μl of pre-heated 0.2% metaphor agarose were pipetted onto filters. After the metaphor agarose had dried, filter pieces were transferred to 0.5 ml sterile tubes containing the hybridization mix. All hybridizations were carried out using the universal eukaryotic FISH probe Euk1209 [104] with 40% formamide for 2 hours at 46°C. After the hybridization step, filter pieces were incubated at 48°C in preheated washing buffer for 10 minutes in sterile 50 ml tubes. Filter pieces were then washed with distilled water and placed into sterile 0.5 ml tubes containing DAPI (2 μg/ml) and incubated for 5 minutes in the dark. Filter pieces were then washed with sterile water and incubated for 2 minutes in 70% ethanol, followed by a 2-minute wash with 100% ethanol. Filters were air dried and mounted on glass slides with a Citifluor/Vectashield mix (4:1) to prevent bleaching. Cells were enumerated under epifluorescence using a Zeiss Axioplan 2 microscope and photographed with a Hamamatsu digital camera.

## Competing interests

The authors declared that they have no competing interests.

## Author’ contributions

AS, VE and TS contributed to project design, collection of data, analysis of data, and drafting of manuscript. WO contributed to drafting the revised manuscript and as well as SF, HWB and MY contributed to collection and analysis of data. All authors have read and approved the final version of this manuscript.

## Supplementary Material

Additional file 1: Figure S1 Rarefaction curves of V4 SSU rRNA-amplicons that were assigned to ciliate genera for all eight samples. Click here for file

Additional file 2: Figure S2 Proportion of rare versus abundant ciliate taxa. The number of detected taxa is opposed to the number of ciliate V4 SSU rRNA amplicons. Click here for file

Additional file 3: Table S1 Number of ciliate V4 SSU rRNA-amplicons in each sample assigned to described ciliate genera. The assigned genus represents the best BLAST hit of assigned amplicons to NCBIs GenBank nucleotide database 187. Click here for file

Additional file 4: Table S2 Keyplayer ciliate taxa with minimum and maximum sequence similarity to known species. Sequence similarities from Genbank BLASTn. Click here for file
