# Supplementary figures and images for: Evidence for isolated evolution of deep-sea ciliate communities through geological separation and environmental selection
Source: BMC Microbiol. 2013 Jul 8;13:150. doi: 10.1186/1471-2180-13-150 (PMC3707832; doi:10.1186/1471-2180-13-150)

## Slide 1
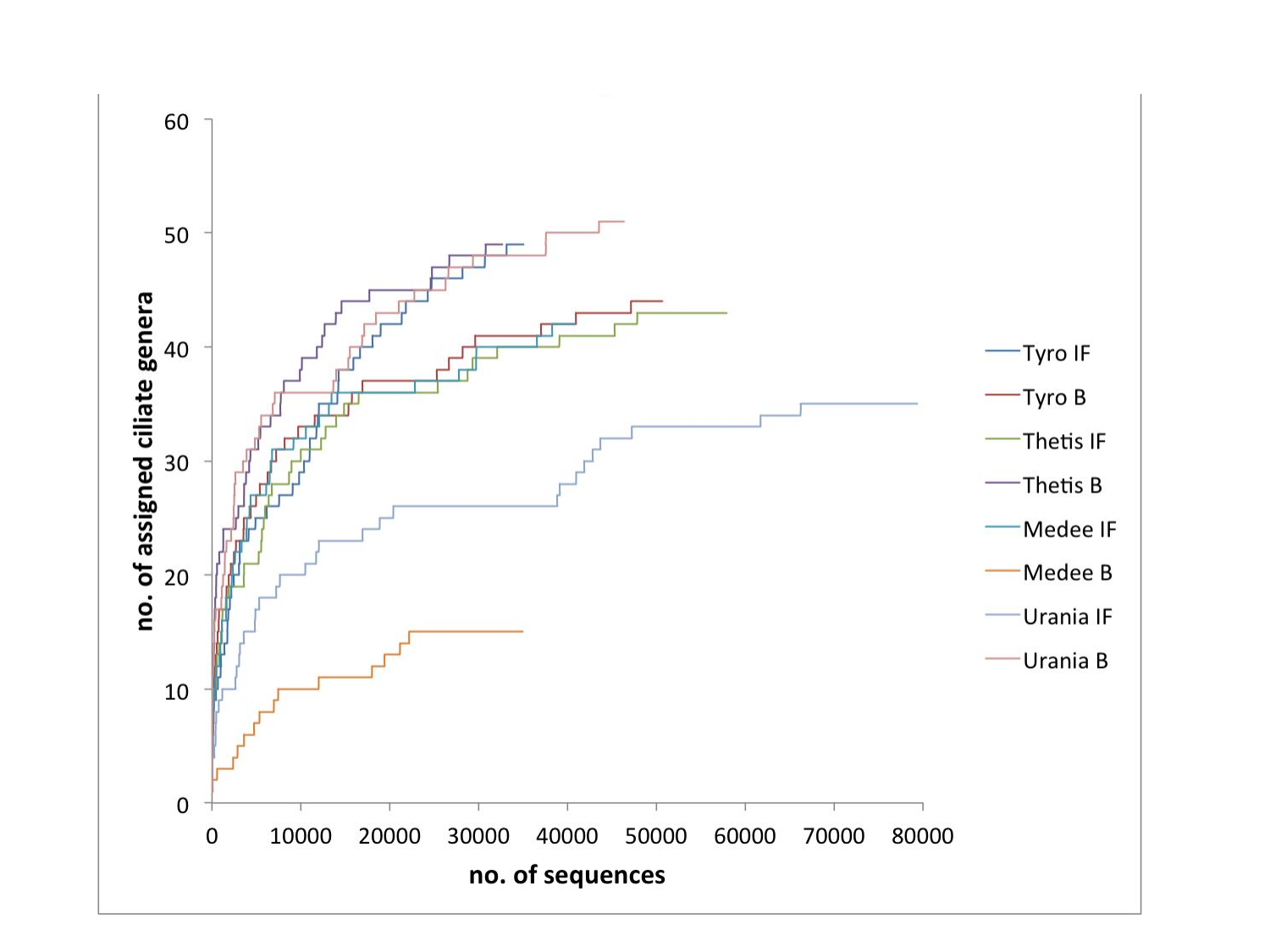

Supplement: Additional file 1: Figure S1 — Rarefaction curves of V4 SSU rRNA-amplicons that were assigned to ciliate genera for all eight samples. [file 1471-2180-13-150-S1.pptx]

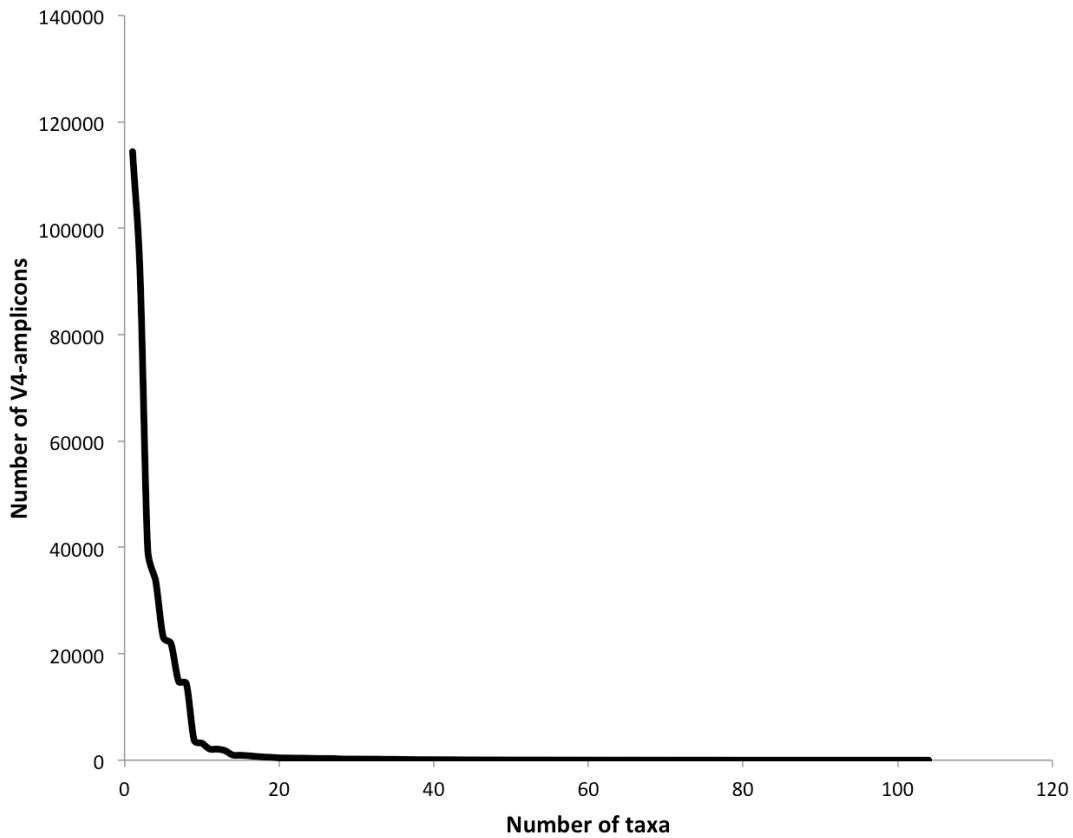

Supplement: Additional file 2: Figure S2 — Proportion of rare versus abundant ciliate taxa. The number of detected taxa is opposed to the number of ciliate V4 SSU rRNA amplicons. [file 1471-2180-13-150-S2.pdf]
